# Polymeric-Calcium Phosphate Cement Composites-Material Properties: *In Vitro* and *In Vivo* Investigations

**DOI:** 10.1155/2010/691452

**Published:** 2010-07-29

**Authors:** Rania M. Khashaba, Mervet M. Moussa, Donald J. Mettenburg, Frederick A. Rueggeberg, Norman B. Chutkan, James L. Borke

**Affiliations:** ^1^Department Oral Biology, Medical College of Georgia, Augusta, GA 30912-1129, USA; ^2^Department Orthopedic Surgery, Medical College of Georgia, Augusta, GA 30912-1129, USA; ^3^Department of Dental Materials, Misr University, 11787 Cairo, Egypt; ^4^Department of Oral Pathology, Cairo University, 11559 Cairo, Egypt; ^5^Department of Oral Pathology, Misr University, 11787 Cairo, Egypt; ^6^Department of Dental Materials, Medical College of Georgia, Augusta, GA 30912-1129, USA

## Abstract

New polymeric calcium phosphate cement composites (CPCs) were developed. Cement powder consisting of 60 wt% tetracalcium phosphate, 30 wt% dicalcium phosphate dihydrate, and 10 wt% tricalcium phosphate was combined with either 35% w/w poly methyl vinyl ether maleic acid or polyacrylic acid to obtain CPC-1 and CPC-2. The setting time and compressive and diametral tensile strength of the CPCs were evaluated and compared with that of a commercial hydroxyapatite cement. *In vitro* cytotoxicity and *in vivo* biocompatibility of the two CPCs and hydroxyapatite cement were assessed. The setting time of the cements was 5–15 min. CPC-1 and CPC-2 showed significantly higher compressive and diametral strength values compared to hydroxyapatite cement. CPC-1 and CPC-2 were equivalent to Teflon controls after 1 week. CPC-1, CPC-2, and hydroxyapatite cement elicited a moderate to intense inflammatory reaction at 7 days which decreased over time. CPC-1 and CPC-2 show promise for orthopedic applications.

## 1. Introduction

There is a high clinical demand for synthetic bone substitution materials, due to drawbacks associated with biological bone grafts. Xenografts are generally associated with potential infections. The same is true for allografts, where there are concerns regarding antigenicity and transmission of infectious diseases in spite of rigorous control on the selection of donors and the preparation of the graft. Thus, interest in synthetic implant materials for bone grafting has been on the rise 

According to Reuger, there are different classes of bone substitute materials which are very prominent in ceramics [1]. The basis of these substitutes is usually calcium phosphate, due to its good biocompatibility because of its similarity to the mineral phase of natural bone tissue [2]. The search for better results has led to modifications in the sintering process by which these materials are formed. These modifications result in multiple forms of calcium phosphate materials, with variations in the calcium-to-phosphate rate and porosity, which can affect both biocompatibility and mechanical resistance. One such variation is calcium phosphate cement (CPC), a term introduced by Groninger et al. [3]. According to these authors, CPC consists of two components, one basic and one acidic which react when mixed with water, producing one or more products with an intermediate acidity. 

Calcium phosphate cements can easily adapt to the shape of bone cavities and defects leading to a close apposition to the host tissue with osseointegrative properties comparable to or better than bulk CPCs [4]. The hardening of the cement, which usually consists of an aqueous solution and one or several calcium phosphates, occurs at a low temperature through a setting reaction that leads to the *in situ *formation of a solid calcium phosphate [5]. A number of CPCs currently are available commercially [6–8]. However, due to their limited compressive strength, they are restricted primarily to nonstress-bearing applications. These include uses in maxillofacial surgery, the repair of cranial defects, and dental fillings [6–8]. In order to improve the mechanical properties of CPCs, a number of researchers have blended polymers with CP cements and met with promising results. Durucan and Brown [9, 10] made *α*-tricalcium phosphate/polylactic acid (*α*-TCP/PLA) and *α*-TCP/polylactic-coglycolic acid (PLGA) blends with a subsequent hydrolysis of *α*-TCP to calcium-deficient hydroxyapatite (CDHA), which showed a modest improvement over the pure cement. Fujishiro et al. [11] added gelatin to their cement formulations, primarily to stabilize the pastes in aqueous solution before it develops adequate rigidity, and found that they were able to get more than a 50% improvement of the compressive strength. They also demonstrated an improvement in mechanical properties by adding rod-like hydroxyapatite and Perovskite (CaTi03) powders to the cements. Miyazaki et al. [12, 13] used a number of polymers including polyacrylic acid (PAA) and polyvinyl alcohol (PVA), to improve the properties of tetracalcium phosphate-dicalcium phosphate dehydrate (TTCP-DCPD) cement. They noted marked increases (up to three-fold) in mechanical properties, but an unacceptable reduction of workability and setting time. Dos Santos et al. [14] reported similar results using sodium alginate and sodium polyacrylate. 

Matsuya et al. [15] reported on the reaction of a less reactive polyacid (the hydrolysis product of 1 : 1 copolymer of methyl vinyl ether and maleic anhydride) with calcium phosphate cement (CPC) or tetra calcium phosphate (TTCP). This commercial copolymer, which is offered in several molecular weights, can be dissolved with hydrolysis of the anhydride groups in water to form the corresponding maleic acid copolymer, polymethyl-vinyl ether-maleic acid. It is known to have a number of nondental applications (hair sprays, surgical adhesives), which suggests potentially favorable biocompatibility for dental and other biomedical uses. The cement forming reaction was significantly faster than that of a water setting CPC but slower than that observed with the mixed powder and polyacryclic acid. The diametral tensile strength and compressive strength of this polymeric calcium phosphate cement (CPC) at 24 hrs and after storage in distilled water were 13.7 MPa and 71 MPa, respectively. 

In light of the preceding discussion, we endeavored to improve the mechanical and physical properties of the cement by adding water soluble polymers during setting. Drawing in results from the literature gave us our candidate polymer components. In the present study, we tested a hypothesis that incorporation of polymeric acids into traditional calcium phosphate cements (CPCs) would produce formulations with improved handling, setting, and mechanical and biologic properties to permit orthopedic applications. The present study tests this hypothesis by measuring the initial and final setting time of two novel CPC formulations derived from a mixture of CPC powder with two aqueous solutions of polymeric acids, polyacrylic acid, and 35% w/w polymethyl-vinyl ether-maleic acid as compared to a commercially available hydroxyapatite cement (Bone Source, StrykerLeibinger Gmb & Co. KG, Freiburg, Germany). The compressive and diametral tensile strength of these two polymeric formulations compared to hydroxyapatite cement. To evaluate comparatively, the cytotoxic properties of the two novel CPC formulations and commercially available hydroxyapatite cement on an osteoblast cell line (ROS17/2.8) and the biocompatibility of these materials following implantation into the subcutaneous connective tissue of rats.

## 2. Materials and Methods

Two calcium phosphate cements and commercial hydroxyapatite cement (Bone Source, Stryker Leibinger Gmb & Co. KG, Freiburg, Germany) were prepared and evaluated (Table 1). The cement formulations were as follows.

- Calcium phosphate cement was derived from a mixture of 60 wt% tetracalcium phosphate, 30 wt% dicalcium phosphate dehydrate, and 10 wt% tricalcium phosphate.
- Two types of aqueous solutions of acids were used for mixing the powder to formulate the calcium phosphate cements.

### 2.1. Preparation of Calcium Phosphate Cement Powder

Tetracalcium phosphate powder was synthesized from a solid state reaction between calcium hydrogen orthophosphate anhydrous (CaHPO_4_) and calcium carbonate, then ground and sieved to obtain an average particle size of 1 *μ*m to 80 *μ*m. Dicalcium phosphate dehydrate powder (DCPD) was obtained from monocalcium phosphate and calcium oxide, which were crushed separately in an agate mortar to obtain an average particle size of 80 *μ*m. Tricalcium phosphate Ca_3_(PO_4_)_2_ was prepared by a crystallization method from aqueous solutions of 0.9 M calcium nitrate (Ca(NO_3_)_2_ · 4  H_2_O and 0.6 M ammonium phosphate (NH_4_)_2_HP0_4_, which were simultaneously mixed. The reaction pH was maintained between 5 and 6 by the addition of ammonia solution. 

The precipitated powder was stored for 24 hr at room temperature, then washed with deionized water and lyophilized. The subsequent calcinations of the resulting powders were obtained at 900°C for over 1 hr. The tetracalcium phosphate, dicalcium phosphate dihydrate and tricalcium phosphate were then mixed at a molar ratio 1 : 1 : 1 in a blender (Dynamics Corporation of America, New Hartford, CT) to form the CPC powder.

### 2.2. Preparation of Aqueous Solution of Liquids

Two types of liquids previously mentioned in Table 1 were mixed with the calcium phosphate powder: 35% (w/w) aqueous solution of polymethyl-vinyl ether-maleic acid was prepared by dissolving 35 grams of polymethyl-vinyl ether-maleic anhydride (PMVE-MA) copolymer (MW 50,000) in 100 mL of distilled water at 60°C for 24 hrs in a shaker incubator. The 35% w/w aqueous solution of PMVE-Ma was mixed with CPC powder to form the polymeric CPC cement. 10% (w/w) aqueous solution of polyacrylic acid (PAA) was prepared (2 mL of PAA solution was added to 2 mL of 10% water).

### 2.3. Preparation of Calcium Phosphate Cements

#### 2.3.1. CPC-1

CPC powder (60 wt% tetracalcium phosphate + 30 wt% dicalcium phosphate dehydrate + 10 wt% tricalcium phosphate). Liquid: 35% (w/w) aqueous solution of polymethyl-vinyl ether-maleic acid.

#### 2.3.2. CPC-2

CPC powder (60 wt% tetracalcium phosphate + 30 wt% dicalcium phosphate dehydrate + 10 wt% tricalcium phosphate). Liquid: 10% (w/w) aqueous solution of polyacrylic acid

### 2.4. Setting Time Measurements

The setting time of the cements were measured according to the International Standard ISO 9917 [16] for dental water-based cement. Ninety seconds after the end of mixing the CPC powders with liquid, the indenter (300 + 5 g in mass, 1 + 0.05 mm in diameter of the needle was carefully lowered vertically on to the surface of the cement and allowed to remain there for 5 s. Initial setting occurs when a 1 mm needle penetrates 25 mm into cement paste. Final set occurs when there is no visible penetration. Each test was repeated five times and the average value was calculated.

### 2.5. Assessment of the Mechanical Properties of the Prepared Cements

#### 2.5.1. Preparation of the Compressive Strength Test Specimens

Steel cylindrical molds with inner diameter of 6 mm and height of 12 mm were used to prepare the cement columns for compressive tests according to the ISO specification no. = 4104 [17] for zinc polycarboxylate cement. The cement pastes were mixed as previously described and inserted into a split mold with a release agent to prevent adherence of the cements. The split mold was covered with a glass plate for 10 min, and then kept undisturbed for another 50 min at 37°C under 100% relative humidity before separation from the mould [15]. The cylindrical specimens were immersed in simulated body fluid (SBF) at 37°C [18] for 30 min, 1 hour, 4 hrs, 24 hrs, 1 week, and 2 weeks before the compressive strengths were measured. 

#### 2.5.2. Preparation of the Diametral Strength Specimens

For the diametral tensile strength test, disc specimens of 6 mm diameter and 3 mm height were prepared for each type of cement [19]. The specimens were prepared as previously described for the compressive strength test

### 2.6. Testing Procedure

The compressive and diametral strength tests of each type of cement were determined after 30 min, 1 hour, 4 hrs, 24 hrs, and 4 weeks storage in SBF using a Universal Testing Machine ( Canton, MA, USA) model no. = 4465 equipped with a 2 KN load cell at a crosshead speed of 0.5 mm/minute^−1^. The compressive strength was measured by dividing the maximum load in compression on the ends of the cylindrical specimens by the original cross-sectional area of the test specimen [20]. The diametral tensile strength was calculated according to the equation (*D* *T* *S* = 2*P*/*D* *T*), where *P* is the applied load, *D* is the diameter of the cylinder, and *T* is the thickness of the specimen. A sheet of filter paper (Whatman Type no. 1, Whatman International, Spring –Field Mill, Maidstone, Kent, England) was placed underneath and another sheet was placed on the top of the specimen during the loading. Each measurement was repeated six times, and the average value was calculated. Quantitative data are presented as mean ± standard  deviation and statistical analysis was performed using a one-way analysis of variance (one-way-ANOVA). A comparison between two means was made using *Tukey's *test, with statistical significance set at *P* < .05.

### 2.7. Cell Culture Experiments

#### 2.7.1. Cytotoxicity Samples

Sample preparation was performed aseptically to prevent the risk of biological contamination during the cytotoxicity testing [21]. Commercial hydroxyapatite cement was prepared according to the manufacturer's instructions. 

Liquids previously mentioned were mixed with calcium phosphate. The powder liquid to ratio of 4 : 1 was selected for the preparation of the three types of cements. This ratio produced good handling characteristics and working time. 

Six discs for each cement (Types I and II CPCs and Hydroxyapatite cement) were fabricated in sterile Teflon molds 5.5 mm in diameter and 3 mm thick. The materials were packed into the mold and allowed to set at room temperature (25°C) before testing. Teflon discs were used as a negative control.

#### 2.7.2. In Vitro Biological Testing

Materials were tested for *in vitro *cytotoxicity in direct contact format (ISO10993) [22] using ROS 17/2.8 osteoblast-like cells.^. ^Cells were maintained in F-12 medium containing 1.1 mM CaCl_2_ (Allied Chemical Corporation, Morristown, NJ), 5% Nu Serum (Collaborative Research, Bedford, MA, USA), 25 mM L-Glutamine, and 125 units/mL penicillin/streptomycin (GIBCO, Grand Island, NY, USA). Twenty-four hrs prior to the addition of the test specimens, the cells were plated at 30,000 cells/cm^2^ in a 24-well format (Costar, Cambridge, MA, USA) in 1 mL of medium per well, then specimens were immediately (<1 min) added to the center of each well and secured such that the sample could not move. The ratio of the surface area of the discs to the volume of medium was within the range of 1.2 mm^2^/mL, as recommended by the International Standards Organization [23]. The cells and specimens were incubated at 37°C for 48 hrs in 5% CO_2_, 95% air to allow attachment of osteoblasts to the bottom of the wells. 

Cellular activity was assessed by measuring mitochondrial succinic dehydrogenase (SDH) activity at several time points to determine if trends in the biological response were observable. Cytotoxicity was initially assessed for 24 hr. After this interval, the specimens were removed from the cell culture, rinsed with sterile phosphate buffered saline (PBS), and then immediately added to a second cell culture (which had been plated with cells 24 hrs earlier). This second culture was incubated for 24 hrs and the mitochondrial activity assessed after incubation (48 hrs reading). The specimens were then removed again, rinsed again, and incubated in cell culture medium without cells for 5 days at 37°C and 5% CO_2_. Finally, the specimens were rinsed again with PBS and added to a third culture that had been plated with cells 24 hrs before. The mitochondrial activity was then assessed 24 hrs later (1 wk reading). 

At each of the three time points (24 hrs, 48 hrs, and 1 wk), the cells were incubated for 24 hrs in the presence of the materials or Teflon discs (negative control) before assessing cellular activity by measuring SDH activity. The SDH activity, indicative of cellular mitochondrial function, was measured by means of the MTT colorimetric assay [24]. Specimens were removed from each well, and the remaining cells were washed carefully with 1.0 mL of phosphate–buffered saline (pH 7.4). A 1 mg/mL MTT solution [3-(4,5-dimethyl–thiazol-2-yl-) 2,5-diphenyl tetrazolium bromide-succinate] was added for 45 min at 37°C, after which the reaction was quenched with the addition of 0.5 mL of 4% Tris-formalin (pH 7.4) for 2-3 min. 

The MTT-formalin solution was removed and the cell monolayer allowed to dry for 5–10 min, then washed in 1.0 mL of water, and any MTT-formazan formed by SDH activity in the cells was solubilized with 6% dimethylsulfoxide–NaOH (0.1 N NaOH in DMSO). An aliquot of the resulting solution was transferred to a 96-well flat-bottomed tray and the optical density was measured at 562 nm (the absorption peak of the formazan) with a microplate reader (MR 5000, Dynatech Laboratories, Embrach-Embraport, Switzerland). The optical density of the blank wells were subtracted from all wells, then the formazan content of each well was computed as a percentage of the Teflon control materials. Differences between groups were determined by ANOVA and Tukey multiple comparison intervals (*α* = 0.05).

#### 2.7.3. Animal Experiments

**(1) Implantation into Subcutaneous Connective Tissue**

Fifty-four ten-week-old male Wistar rats (Ratus norvegicus albinus) weighing 200–250 grams obtained commercially and given standard pellets and water *ad libitum *were used for the implantation study. IACUC guidelines for the care and use of laboratory animals were observed.

The animals were anesthetized by intramuscular administration of rodent anesthesia cocktail (1.5 mL ketamine HCl (100 mg/mL), 1.5 mL xylazine HCl (20 mg/mL) and 0.5 mL acepromazine (10 mg/mL) 0.2–0.5 mL per 100 g of animal weight and stabilized on a surgical table. For the implantation of the CPCs, the backs of the rats were shaved, washed and disinfected with 5% iodine solution. Three longitudinal incisions of about 1 cm in length were made through the full thickness of the skin using a number 15 blade in a head-to-tail alignment orientation. Subsequently, lateral to the incisions subcutaneous pockets were created by blunt dissection with scissors. The experimental materials CPC-1 and CPC-2 were mixed at a powder to liquid ratio of 4 : 1 and the commercial hydroxyapatite cement was mixed according to the manufactures' instructions. Cements were then packed in a cylindrical mould so that the paste samples were 4.7 mm in diameter and 10 mm in length. The mould was made by cutting the front portion of a 1 cm^3^plastic syringe. A force of approximately 0.5 MPa was applied for packing the cement paste into the moulds. After smoothing the surface of the cement on the open side, cements were inserted randomly into each subcutaneous pocket. These procedures were done quickly so that the cement was implanted 1 min after mixing. Finally, the incisions were carefully closed with 3/0 silk sutures.

Groups of six animals per material were sacrificed at 7, 30 or 90 days after the implantation procedures.

After the experimental periods, all animals from each group were anesthetized, the dorsal skin was shaved and disinfected, and the implants together with their surrounding tissue were removed in blocks. Animals were killed by an overdose of anesthetic immediately after removal of tissue samples.

**(2) Histological Preparation**

The subcutaneous sites containing the implants were carefully excised from the surrounding tissue and immersed in buffered 10% formalin fixative solution for 48 hrs. The biopsies were processed for paraffin embedding. Six micrometer serial sections were obtained and stained with hematoxylin and eosin. The stained sections were assessed by light microscopy (Carl Zeiss, Oberkachen, Germany).

Tissue reactions such as inflammatory response, thickness of fibrous capsule adjacent to the implanted material, and the presence of macrophages and giant cells were described based on a subjective single-blinded evaluation, as previously reported in [25]. The histological event inflammatory reaction was recorded as: none, mild, moderate, and severe, according to the inflammatory cell number present close to the materials. To grade the inflammatory reaction, five high-power magnification fields were selected for all slides. The cell number was recorded with a grid per high-power field. The thickness of fibrous capsule formation was measured for each specimen using a light microscope (Diastar, Cambridge Instruments, Buffalo, NY, USA) adapted to a video-camera (DXC-107A/107P; Sony Electronics, Inc, Tokyo, Japan) and to a microcomputer and software (Mocha, Jandel Scientific, San Rafael, CA, USA). Fibrous capsule formation thinner than 150 *μ*m was scored as 1-thin. Fibrous capsule formation thicker than 150 *μ*m was recorded as 2-thick. Macrophages and giant cells presence was recorded as 1-none, 2-mild, and 3-moderate, according to the number of these specific inflammatory cells present close to the calcium phosphate materials, indicating a persistent connective reaction against the implants. The experimental materials were regarded as biocompatible if the intensity of connective tissue reaction decreased over time. Consequently, to be considered biocompatible, at 90 days the connective tissue surrounding the implant must show a thin fibrous capsule formation as well as an absence of inflammatory reaction and/or macrophages/giant cells. On the other hand, the material was considered nonbiocompatible when a persistent inflammatory reaction occurred related to macrophages and giant cells with a thick fibrous capsule development even at 90 days after implantation [25]. The scores obtained from the histological assessment of sections were submitted to the statistical analysis of Kruskal-Wallis complemented by Mann-Whitney test taking into account a level of significance5%.

## 3. Results

### 3.1. Setting Time

**Figure 1:**
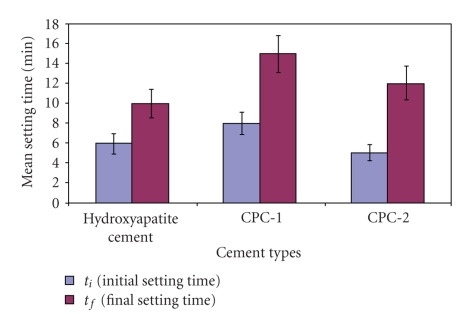
Histogram showing the mean initial and final setting times (in min) of hydroxyapatite cement and the two polymeric calcium phosphate cements (CPCs).

The results of the initial and final setting times are presented in Table 2 and illustrated in Figure 1. After mixing the powder and liquid components together, the two polymeric cements One parameter to estimate the setting of the cement is the setting time (t_i_: initial setting time and t_f_: final setting time) of the cement paste. The setting time should be within a limited time range; 3 min <t_i_ < 8 min and t_f_ < 15 min [26]. As illustrated in Figure 1, under the conditions used, for polymeric calcium phosphate cements (CPC-1 and CPC-2) such optimal settings were achieved.

### 3.2. Mechanical Properties

**Figure 2:**
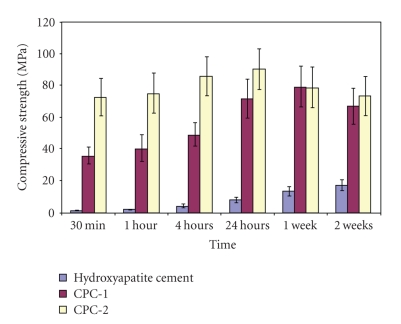
Histogram showing the mean compressive strength of hydroxyapatite cement and the two polymeric CPCs in MPa.

**Figure 3:**
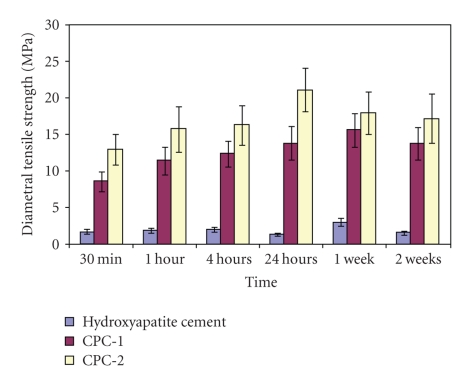
Histogram showing the mean diametral strength of hydroxyapatite cement and the two polymeric CPCs in MPa

The mean values of compressive strength and diametral tensile strength of hydroxyapatite cement (control group) and the two polymeric calcium phosphate cements (CPC-1 and CPC-2) are listed in Tables 3 and 4 and illustrated graphically in (Figures 2 and 3). 

#### 3.2.1. Compressive Strength

At all the storage periods, from 30 min to 2 weeks, the compressive strength values of polymeric calcium phosphate cement (CPC-2) derived from 10% (w/w) aqueous solution of PA acid were higher than that of hydroxyapatite cement (control) and CPC-1 cement ( *P* < .05). When comparing the effect of storage time on the compressive strength of the individual cements, the compressive strength of hydroxyapatite cement significantly increased from 30 min (1.38 MPa) to 24 hrs (8.08 MPa), and then significantly increased up to 2 weeks (17.41 MPa). 

The mean value of compressive strength of (CPC-1) polymeric CPC derived from 35% aqueous solution of (PMVE-Ma) significantly increased from 30 min (35.85 MPa) to 24 hrs (71.68 MPa), then slightly decreased at the end of the second week (67.12 MPa). The mean value of compressive strength of (CPC-2) polymeric CPC derived from PA significantly increased from 30 min (72.68 MPa) up to 4 hrs (86.05 MPa) then decreased at the end of the second week (75.59 MPa).

#### 3.2.2. Diametral Tensile Strength

At all the storage periods from 30 min to 2 weeks, the mean diametral strength values of polymeric calcium phosphate cement (CPC-2) derived from 10% w/w aqueous solution of PA were higher than that of hydroxyapatite cement and (CPC-2) cement (*P* < .05). When comparing the effect of storage time on the mean values of diametral tensile strength, hydroxyapatite cement showed the highest diametral strength values at the end of the first week (2.99 MPa), then slightly decreased at the end of the second week (1.51 MPa). The mean value of diametral tensile strength of (CPC-1) polymeric CPC significantly increased from 30 min (8.57 MPa) up to one week (16.63 MPa), then significantly decreased at the end of the second week (13.77 MPa). The mean diametral tensile strength value of (CPC-2) polymeric CPC cement significantly increased from 30 min (12.94 MPa) to 24 hrs (21.12 MPa) then significantly decreased at the end of the second week (128.38 kg/cm^2^)

### 3.3. Cell Culture (MTT Assay)

**Figure 4:**
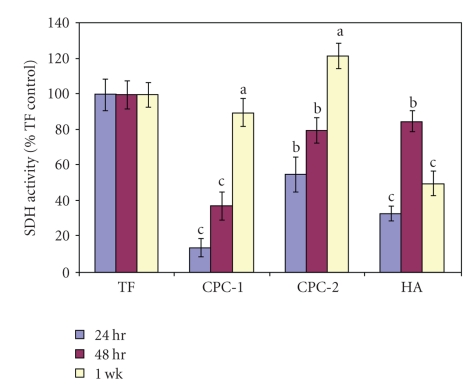
Mitochondrial suppression induced by CPC-1, CPC-2, and commercial HA. Cytotoxicity was evaluated at three time points 24 hrs, 48 hrs and after 1 wk. Succinic dehydrogenase activity was measured and expressed as a percentage of Teflon Controls (defined as 100%). There were six replicates per condition. Different letters indicate a statistically significant difference between the materials (ANOVA, Tukey intervals *α* = 0.05)

Aging significantly influenced the cytotoxicity of the materials, which became less cytotoxic over time (Figure 4). Overall, our results suggested significant differences between materials, and that cytotoxicity generally decreases with time. Type I CPC was cytotoxic in the initial 24 hrs of testing, but SDH activity had significantly (*P* < .05), increased (>90%) and was statistically equivalent to the negative Teflon Control after 1 wk. At 24 hrs, CPC-2 suppressed SDH activity by >55% relative to the Teflon Control, and then showed some tendency toward an increase in SDH activity with time (48 hrs), although differences were not statistically significant. At 1 wk, CPC-2 was significantly higher than the Teflon Control. Hydroxyapatite cement suppressed SDH activity by 33%, and 85% relative to the Teflon negative control after 24 hrs and 48 hrs, respectively, and then showed a relapse by 35% after 1 week.

### 3.4. Implantation into Subcutaneous Connective Tissue of Rats

The relationship among the periods of evaluation and the connective tissue reaction (i.e. the presence of inflammatory cells, formation of fibrous capsule close to the implants, and the presence of macrophages and giant cells) are given in Table 5.

#### 3.4.1. Seven Days

**Figure 5:**
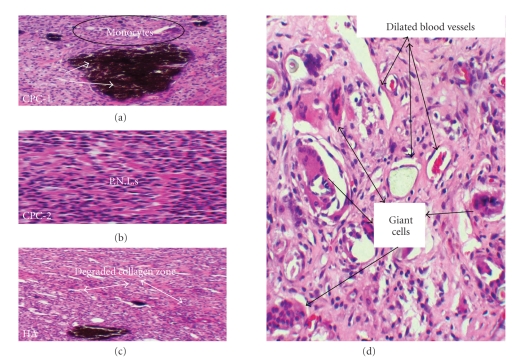
Tissue reactivity to implanted materials at 7 days. (a) this shows mild inflammatory reaction mediated by mononuclear monocytes in response to the implanted CPC-1 (arrows). (b) Implanted CPC-2 at 7 days shows a moderate inflammatory reaction mediated by mononuclear cells. (c) Implanted HA at 7 days exhibits an area of collagen degradation (arrows). (d) Example of the inflammatory reaction characterized by small dilated blood vessels and presence of giant cells observed in response to all the implanted materials (CPC1 shown) H&E X200.

A similar histological characteristic regarding the presence of macrophages and thickness of the fibrous capsule was demonstrated for all experimental groups. Most of the samples exhibited thick capsules. Also, in these samples, a mild presence of macrophages adjacent to the implanted materials was observed (Figure 5 (a)). However, for CPC-1, CPC-2, and HA, a moderate inflammatory reaction was demonstrated in 5, 4, and 3 samples, respectively. This inflammatory response mediated by mononuclear cells (Figure 5 (b)) was also characterized by the presence of a number of small congested and dilated blood vessels, multinucleated giant cells, local edema, and collagen degradation (Figures 5(c) and 5(d)). Only 1 sample of each experimental group exhibited a severe inflammatory reaction (Table 5). 

#### 3.4.2. Thirty Days

**Figure 6:**
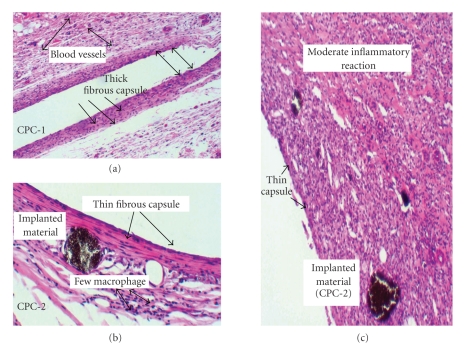
Tissue reactivity to implanted materials at 30 days. (a) Note the thick fibrous capsule (arrows) and the small dilated blood vessels in response to the implanted material (CPC-1) (arrows). (b) CPC-2 at 30 days demonstrates a thin fibrous capsule (arrows) and few macrophages adjacent to the implanted material. (c) Area showing a moderate inflammatory reaction and a thin fibrous capsule as seen in CPC-2 and HA implanted materials (CPC-2 shown). H&E X200.

For CPC-1, CPC-2, and HA, a mild inflammatory reaction was observed for 3, 4, and 3 samples, respectively (see Table 5). Most of these samples exhibited a thin fibrous capsule with a few macrophages adjacent to the implanted calcium phosphate materials (Figure 6 (a)). Those samples in which a persistent moderate inflammatory reaction mediated by mononuclear cells occurred, a thick capsule with several small blood vessels were observed (Figure 6 (b)). Only one sample each of CPC-1 and HA exhibited a moderate presence of macrophages adjacent to the implanted materials (Figure 6 (c)).

#### 3.4.3. Ninety Days

**Figure 7:**
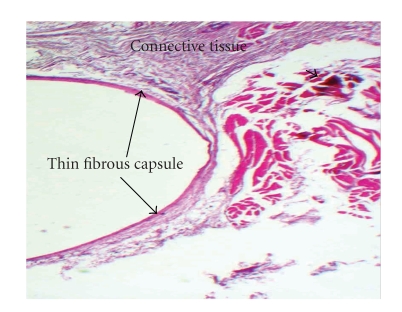
Section of tissue from area of CPC-2 implantation which demonstrates a thin fibrous capsule and a normal connective tissue denoting complete healing at 90 days. Note that this reaction is typical of all experimental implanted materials at 90 days H/E X200.

Connective tissue repair was observed for all experimental materials. However complete healing occurred only in 5 samples of those calcium phosphate cements in which thin fibrous capsule and lack of inflammatory reaction were observed (Figure 7). One of each samples, CPC-1 and HA exhibited a persistent inflammatory reaction. In both samples, a zone of dispersed cement fragments (displacement of the components of the experimental material) was observed at a great distance from the implants, triggering a noticeable chronic connective tissue reaction. For HA, two samples presented with a thick fibrous capsule adjacent to the implanted material. In these samples, a mild presence of macrophages persisted. 

Despite the more intense connective tissue reaction observed for HA when compared to CPC-1 and CPC-2, the statistical analysis of Kruskal-Wallis demonstrated that there was no difference among the experimental groups regarding the histological features observed. According to the periods of evaluation, a statistical significant difference was determined only between 7 and 90 days postoperative time for all experimental groups (Mann-Whitney test, *P* < .05). For CPC-1, a significant difference in the presence of macrophages and giant cells was observed when the periods of 7 and 30 days were compared to the period of 90 days

## 4. Discussion

The advent of calcium phosphate bone cements (CPCs) is considered as a remarkable development in the field of bone repair materials. Calcium phosphate bone cements (CPCs) have been emerging as a new family of resorbable bone substitutes since the mid 1980's. However, problems such as poor mechanical properties and relatively long setting times which limits their clinical application are still encountered. Therefore, it is the ongoing objective of materials development to improve (CPCs) and prepare compositions with adequate mechanical properties and suitable setting times. In this paper, we report the development of a calcium phosphate bone cement suitable for orthopedic applications with Tetracalcium phosphate (TTCP), dicalcium phosphate dihydrate (DCPD), and Tricalcium phosphate Ca_3_(P0_4_)_2_ as ingredients mixed with aqueous solutions of modified polyacrylic acid (PA), polymethyl-vinyl ether-maleic acid (PMVE-Ma) to improve the physicomechanical properties of CPCs. All these selected materials are of medical grade, commercially available, and have a well established compatibility. 

An essential parameter considered before mixing these essential components is to sieve each one separately up to 80 microns, as reduction of particle size was found to produce a substantial decrease of the setting time and accelerate the hardening of the cement without significantly affecting the final strength attained [27]. Particle size can also influence different characteristics of materials, for example an increased surface area (smaller particle size) can lead to greater dissolution during the setting reaction [28]; decreased working and setting times [29]; higher compressive strength (CS); and higher diametral tensile strength (DTS) [30]. 

Other essential criteria were also considered in relation to the molecular weight and concentration of the aqueous solutions used for mixing the powder, as higher molecular weights tend to result in cements with shorter setting times and higher compressive, diametral, and biaxial flexural strengths than their lower molecular weight counterparts [31]. 

The polymethyl-vinyl ether-maleic anhydride (PMVE-Ma) is a commercial copolymer offered in several molecular weights and can be dissolved by hydrolysis of the anhydride group in water to form the corresponding maleic acid copolymer (polymethyl-vinyl ether-maleic acid). 

This copolymer has already a number of nondental applications, in hair sprays and surgical adhesives which suggest potentially favorable biocompatibility for dental and other biomedical uses [15]. Because it is difficult to form workable cements from highly concentrated solutions of PMVE-Ma due to their high viscosities, concentrations above 30% could not be investigated. However, aqueous solutions of higher concentrations are feasible using lower molecular weight PMVE-Ma. In the present work, PMVE-Ma (50,000 molecular weight) was used in an aqueous solution of 35% w/w [15]. PAA was used as an aqueous solution and diluted with distilled water to obtain 10% w/w [32]. 

The addition of poly (acrylic acid), a water soluble polymer, to CPC would be expected to result in a reaction akin to the setting reaction in glass ionomer cements, which would remedy some of the problems. In addition, the basicity of TTCP is also expected to result in a rapid setting time via the neutralization reaction, which in turn would result in crosslinking of the polymers. In order to optimize the powder/ liquid ratio of the mixing powder, preliminary testing of various powder mixture ratios was performed until an optimal ratio of 4 : 1 was obtained. 

### 4.1. Setting Time

There are two stages identified with the setting of CPCs, the initial setting time which denotes the end of the workability of the putty after wetting, and the final setting time which indicates hardening of the set mass [33]. The manipulation of the setting times of CPCs is significant as they should meet the requirements of surgical procedures. The initial setting should be easily adjusted so as to allow a sufficient time gap for shaping and filling. After the filling, it is not advisable to disturb the set cement until its hardening because any mechanical strain will produce cracks and adversely affect the strength. Therefore, CPCs require the shortest possible final setting time so that the wound closure is not delayed. The presented results suggest that calcium phosphate cement mixed with polymeric liquids had a positive influence on the material setting properties and the workability. CPC-1 mixed with PMVE-Ma acid (Table 2) exhibited initial and final setting times of, respectively, 8 and 15 min. These results coincide with Dricssens et al. [26] as recommended for orthopedic applications. As for CPC-2 mixed with modified polyacrylic acid, this cement showed initial and final setting times of 5 and 12 min. Under the operation procedures, the setting time should be within a limited time range of 3 min <t_i_ < 8 min and t_f_<15 min [26]. Low differences between *t*_*i*_ and *t*_*f*_ improved the time scale of the operation procedure.

### 4.2. Mechanical Properties

Results of the compressive and diametral tensile strength values of the newly formulated polymeric calcium phosphate cements are shown in Tables 3 and 4. These are denoted as outstanding and improved values when compared to hydroxyapatite bone cement (control group). 

In this study, the reaction between CPC powder and 35% w/w (PMVE-Ma) aqueous solution resulted in a polymeric CPC-I with compressive strength of 71.68 MPa and diametral strength of 13.8 1 MPa 24 hrs after mixing. These results are in accordance with Matsuya et al. [15]. At the end of the first week maximum strength values were reached. 

The cement prepared from CPC powder and modified polyacrylic acid (CPC-2) had a compressive strength value of 90.57 MPa and diametral tensile strength of 16.3 MPa, 4 hrs after mixing and reached its maximum value at the end of 24 hrs. These high early strength values suggest that the polymerization reaction is responsible for increasing of the initial strength. 

For the commercial hydroxyapatite cement, the set cement has sufficient mechanical strength (17.41 MPa) comparable to that of trabecular bone. These features make this material suitable for applications as bone filler. 

It was apparent from mechanical testing that compressive strength (CS) and Diamertral Tensile Strength (DTS) were influenced and positively correlated to the composition of the cement powder. These results show that polymeric liquids improved the mechanical properties of cements, most likely favored a better distribution of the mechanical load, and therefore had the consequent greater values of mechanical strength with respect to the control cement. Dramatic improvements in mechanical properties were observed with polyacrylic acid (PA) and polymethyl-vinyl ether-maleic acid (PMVE-Ma) composite cements. Both systems exhibited similar behavior. Polymeric calcium phosphate cements set as the result of two reactions, the typical acid-base reaction, and a polymerization reaction of polymer monomers. The resulting structure is a cement matrix reinforced by the interpenetrating chains of the polymer. These results suggest that the cement-forming reaction of CPC-1 in the presence of (PMVE-Ma) acid, TTCP mainly reacts with the carboxylic acid groups of PMVE-Ma by ion exchange or an acid base reaction. This setting mechanism was previously described in other types of polymeric calcium phosphate cements [13]. The amorphous reaction product, that is, the polysalt derived from the reaction of PMVE-Ma with the CPC powder, forms a cement matrix analogous to that formed in polycarboxylate or glass ionomer cements. The filler phase of the polymeric calcium phosphate cements consists of unreacted TTCP and DCPA. The role of both TTCP and DCPA in this type of polymeric cement may be that of reactive filler. 

The following formulation is proposed for the cement forming reaction in the polymeric calcium phosphate cement where R-COOH represents PMVE-Ma

(1) $$\begin{array}{c} \text{R-COOH}\to \text{R-}{\text{COO}}^{-}+{\text{H}}^{+} \end{array}$$

(2) $$\begin{array}{c} {\text{Ca}}_{4}{\left({\text{PO}}_{4}\right)}_{2}\text{O}+2\text{H}\to 4{\text{Ca}}^{2+}+2{{\text{PO}}_{4}}^{3-}+{\text{H}}_{2}\text{O}, \end{array}$$

(3) $$\begin{array}{c} 2\text{R-COOH}+{\text{Ca}}_{4}{\left({\text{PO}}_{4}\right)}_{2}\text{O}\to \text{R-COO-Ca-COO-R}\\ \;\;\;\;\;\;\;\;\;\;\;\;+\;{\text{Ca}}_{3}{\left({\text{PO}}_{4}\right)}_{2}+{\text{H}}_{2}\text{O}, \end{array}$$

(4) $$\begin{array}{c} \begin{array}{cc} & {\text{Ca}}_{4}{\left({\text{PO}}_{4}\right)}_{2}\text{O}+\;2{\text{Ca}}_{3}{\left({\text{PO}}_{4}\right)}_{2}+{\text{H}}_{2}\text{O}\to \;\\ & \;\;\;\;\;\;\;\;\;\;\;\;\;\;{\text{Ca}}_{10}(4\;{\left({\text{PO}}_{4}\right)}_{6}{\left(\text{OH}\right)}_{2}. \end{array}\; \end{array}$$

Calcium ions are released from tetracalcium phosphate powder by the dissolution/neutralization action of hydrogen ions from PMVE-Ma, as shown in formulas (1) and (2). The Ca^2+^ ions from ionic cross links between the PMVE-Ma molecules of the liquid component in the cement mixture are shown in the reaction (3). The phosphate ions released in reaction (2) remain in the cement matrix until the cement is immersed in storage medium, where they can react with calcium and phosphate ions released by hydrolysis of residual tetracalcuim phosphate to produce hydroxyapatite, as shown in formula (4). 

The effect of the molecular weight of PMVE-Ma present in the liquid component is likely to have influenced the compressive strength by virtue of the reinforcement that can be provided by the elongated chains of the polymer matrix. The high molecular weight (50,000) of the PMVE-Ma, in addition to the highly branched structure allows it to bridge numerous crystallites and engage in intermolecular entanglements, providing strengthening mechanisms. 

Nevertheless, the improved diametral tensile strength (DTS) of PMVE-Ma composites (Table 4) clearly suggests the polymer's ability to absorb energy and bridge across multiple crystallites. The strengthening effect of polymeric liquid relates to the fact that in brittle materials, the tensile stress is indirectly controlled by fracture processes initiated from preexisting distributions of flaws and the reduction of porosity related to the polymer addition which can contribute to a significant increase of the strength. Also, the presence of a polymeric, second phase in the cement, although in a small percentage, can result in an increase of the crack propagation resistance. In addition, to the positive effect on diametral tensile strength of using CPC powders having smaller-sized particles. These results are in accordance with Matsuya et al. [15]. 

The cement forming reactions of CPC-2 in the presence of polyacrylic (PA) acid are both neutralization and chelation. The neutralization process occurs via the dissociation of the H^+^ ions from the Poly acrylic acid subunits, which in turn react with the basic TTCP thus enhancing the dissolution of TTCP. As a result, Ca^2+^ ions then participate in a cross linking reaction resulting in the bridging of acrylic acid subunits [34]. The resulting structure is a more tightly crosslinked matrix reinforced by the interpretating chains of the polymer, increasing compressive strength. Also, the diametral strength of CPC-2 cement composites was significantly enhanced due to entanglement of the polymer chains in the cement matrix and the reduction of crack propagation during failure as a result of chain pullout, increasing mechanical strength. 

The improvement in mechanical properties in both cement composites can also be attributed to the powder to liquid (P/L) ratio. It is well-known that the P/L ratio affects the final mechanical properties of CPC, with an increased P/L ratio leading to superior mechanical properties and a concomitant reduction in working time and paste workability [13, 35–37]. The increase in mechanical strength due to an increase in P/L ratio is correlated with a lower overall porosity of the CPC [36, 37]. While P/L ratio may play a role, the polymer's chemical structure and the long chain effects are equally important.

### 4.3. Cell Culture Experiments

Although mechanical and physical properties are of great concern for bone replacement materials, biocompatibility is another critical issue. The cytotoxicity of the two formulations of calcium phosphate cements and commercial Bone Source hydroxyapatite cement were comparatively evaluated regarding the effect on cell proliferation and viability using the ROS17/2.8 osteoblast-like cell line originating from rat bone sarcoma. These cells are well characterized, reproducible, and serve as a good initial screening model for *in vitro *evaluation of the biocompatibility of bone cements [38–40]. The MTT assay, originally introduced in1983 by Mosmann [41], is regarded as a sensitive method for the evaluation of the cytotoxicity of biomaterials [42, 43]. This assay measures cell proliferation and survival via mitochondrial dehydrogenase activity, and is known to be easy, efficient, and reliable [41, 44]. The value of this type of study design is that cytotoxicity of the materials can be observed under identical conditions by allowing direct contact of the cells with the tested materials [42]. This method also provides verification of degradation and/or the affect of dissolution products on cell viability and proliferation. In this way, this method can replicate more closely the physiological situation *in vivo*. The MTT assay showed that CPC-2 was the most biocompatible material evaluated. The high biocompatibility presented by this material and stimulation of the metabolic activity of the cells in culture by CPC-2 after 1 week is not easily explained and warrants further investigation.

Polymethyl-vinyl ether-maleic anhydride (PMVE-Ma) is a commercial copolymer offered in several molecular weights and can be dissolved by hydrolysis of the anhydride group in water to form the corresponding maleic copolymer (polymethyl-vinyl ether-maleic acid). The results of cytotoxicity testing showed that CPC-1 was initially severely cytotoxic to mitochondrial activity, but exhibited reduced cytotoxicity over time. Therefore, it is possible that the initial suppression of activity was caused by release of native mass from the materials (unreacted polyacrylic acid). The polyacrylic acid has a low pH and may leak gradually to the culture cells causing this severe cytotoxicity. Over time, however, it is possible that as cytotoxic elements were leached from the material they either complexed with other molecules in the medium or broke down, in each case rendering it more biocompatible. 

Hydroxyapatite (Bone Source) cement is a mixture of CPC and an aqueous solution of phosphoric acid. Our results showed an increase in SDH activity by 48 hrs followed by a relapse after 1 week which suggests an increase in release of some material components that did not occur initially, rendering the material more cytotoxic over time. The results indicated are in agreement with Rossa et al. [45] whom reported cytotoxicity of several formulations of calcium phosphate cements due to the phosphoric acid present in the liquid phase of the Hydroxyapatite (Bone Source) formulation. The relapse in SDH activity could be explained on the basis of: (a) the low pH value of the phosphoric acid. may have led to a greater change in the pH of the culture medium, resulting in more cellular damage (represented by reduced viability), and (b) the calcium phosphate particles in the cement paste rapidly dissolved during the initial setting reaction up to a complete consumption of the acid; therefore, the calcium and phosphate ion concentrations within the cement liquid are dramatically increased, which may explain the increase in cytotoxicity.

### 4.4. Implantation into Subcutaneous Connective Tissue of Rats

Despite the difference between the materials observed *in vitro*, there was no statistically significant difference between all groups regarding the *in vivo *biocompatibility. As previously reported, the surgical trauma caused during the implantation may elicit an inflammatory reaction at short time implantation [46]. However, it has also been reported that the slow acid/base reaction that occurs in calcium phosphate cements causes the maintenance of the low pH of the material for extended periods of time [47]. 

Therefore, the persistently low pH of the set calcium phosphates in contact to the connective tissue seems to play an important role in the local connective tissue damage, following the implantation procedure. As the connective tissue reaction decreased with time, it may be suggested that the edema adjacent to the set cements might neutralize the effects of the toxic low pH. On the other hand, the moist environment (edema) interferes with the cement setting. Therefore, most of the leached components of the material including soluble ions or molecules and insoluble wear debris and fragments may be displaced at a distance from the implantation site to trigger the moderate to intense inflammatory response observed at the short time evaluation (1 wk). In conclusion, the initial inflammatory response may be elicited by many different irritant factors. 

In the present investigation, it was shown that the intensity and area of the inflammatory reaction decreased with time. However, some samples each of CPC-1 and HA exhibited macrophages and a few giant cells adjacent to the implanted material even at the longest period of evaluation. The more severe inflammatory response to CPC-1 and HA may have been caused by the toxicity of the unreacted polyacrylic acid leaching gradually to the surrounding tissue also for HA cement this could be attributed to unreacted phosphoric acid present in the liquid phase. In these samples, zones of cement particles were dispersed from the implantation site.

Previous *in vivo *studies demonstrated that large granules of calcium phosphate ceramics become encapsulated by a fibrous tissue [48]. In contrast, small particles evoke an intense localized inflammatory reaction, characterized by the presence of multinucleated giant cells and macrophages which in turn release inflammatory kinins like fibroblast growth factor and platelet derived growth factor which influence fibroblast behavior and subsequently induce thickening of the fibrous capsule [49–51]. Similar histological features were observed in the present investigation suggesting that the cement particles, released into the wet environment after the implantation procedure, triggered a persistent local chronic inflammatory response. However, it may be speculated that soluble calcium phosphate fragments released into the connective tissue, might be removed by local lymphatic drainage. This hypothesis should explain why the inflammatory reaction decreased with time and the connective tissue healing occurred for all experimental materials at 90 days following the implantation. 

One each of samples CPC-1 and HA presented a moderate inflammatory reaction. In these samples, macrophages and giant cells were observed engulfing globules and fragments released from the implanted materials. Consequently, it may be suggested that large particles of calcium phosphate were not removed by the local lymphatic drainage or digested by macrophages and giant cells. Consequently, the calcium phosphate particles that remained in the connective tissue may have triggered the persistent chronic inflammatory reaction, which did not allow complete local connective tissue healing. 

The comparison among the data obtained from the *in vitro *and *in vivo *tests in the present investigation clearly demonstrate that both the time of elution and material tested influenced cell respiratory activity. The newly formulated CPC-1 and CPC-2 became less cytotoxic over time. In contrast, the cytopathologic effect evidenced by the commercially available HA increased with time. For the implantation methodology, the leachable components of calcium phosphate cements contributed to the noticeable initial aggressive effects of these materials. However, with time, connective tissue healing occurred characterizing the *in vivo *biocompatibility of these materials. Therefore, it is reasonable to assert that differences in the methods used to evaluate the biocompatibility of biomaterials may result in contradictory data as with results obtained for the commercial HA cement. 

The controversy between *in vitro *and *in vivo *studies to evaluate the cytotoxic effects and biocompatibility respectively of biomaterials has been clearly demonstrated when the results of such investigations are compared [52, 53]. The lack of correlation between *in vitro *tests and clinical experience is probably related to many factors. The duration of *in vitro *tests is probably a major factor since most *in vitro *tests are relatively short time (3–7) days in duration. As stated before above, an *in vitro *study should never be considered a substitute for a clinical or experimental *in vivo *evaluation. On the basis of our *in vitro *observations, we conclude that the two formulations of calcium phosphate cements tested CPC-1 and CPC-2 were comparable to the commercially available positive control. Accordingly, the *in vivo *observations suggest that all calcium phosphate cements evaluated may be considered as biocompatible following implantation into the connective tissue of rats and appear safe to use next to soft tissues. 

The two polymeric CPCs (CPC-1and CPC-2) formulated in the first part of the study, demonstrated reasonable setting times and high mechanical properties. By virtue of these characteristics, coupled with their *in vitro *and *in vivo *biocompatibility, CPC-1 and CPC-2 have properties that show promise for orthopedic applications. CPC-1 and CPC-2 should be further investigated in different *in vivo *models for specific applications such as vertebroplasty

## Figures and Tables

**Table 1:** Composition of the materials used in this study.

Material	Composition	Trade name	Manufacturer
Calcium hydrogen orthophosphate anhydrous			Mallinckrodt Baker, Inc. Phillipsburg, NJ, USA
Calcium carbonate			Mallinckrodt Baker, Inc. Phillipsburg, NJ, USA
Monocalcium phosphate monobasic (MCPM)		Calcium Phosphate Monobasic	Sigma Chemical Laboratories, St. Louis, MO, USA
Calcium oxide			Adwic Laboratory Chemicals Cairo, Egypt
Calcium nitrate	Ca (NO3)_2_·4H_2_O FW 236.15		Sigma Chemical Laboratories, St. Louis, MO, USA
Ammonium phosphate	(NH_4_)_2_HPO_4_ FW 132.06		Sigma Chemical Laboratories, St. Louis, MO, USA
Polymethyl-vinyl ether-maleic anhydrate copolymer (PMVE-Ma)			Sigma Chemical Laboratories, St. Louis, MO, USA
Polyacrylic acid	*M* = 450 kDa; Water content: 2.9 wt%		Sigma Chemicals, St. Louis, MO, USA
Bone Source Classic	Self-setting osteoconductive hydroxyapatite (HA)		Stryker Leibinger Gmb & Co. KG, Freiburg, Germany

**Table 2:** The Initial (I) and Final (F) setting times (in min) of hydroxyapatite cement and the two polymeric calcium phosphate cements (CPCs) (values are *m* *e* *a* *n* *s* ± *S* *D*; *n* = 5).

Type	Initial setting time (min)	Final setting time (min)
Hydroxyapatite cement (Control group)	6 ± 0.477	10 ± 1.40
CPC-1	8 ± 0.95	15 ± 2.5
CPC-2	5 ± 1.40	12 ± 1.7

**Table 3:** Mean compressive strength and standard deviation of hydroxyapatite cement and the two polymeric calcium phosphate cements (CPCs) [the values in parenthesis are in MPa].

Cement Types		30 min	1 hrs	4 hrs	24 hrs	1 week	2 weeks
Control Hydroxyapatite		(1.38) ± 0.18	(1.90) ± 0.30	(4.13) ± 0.70	(8.08) ± 0.90	(13.41) ± 2.10	(17.41) ± 2.90

Calcium phosphate Cements (CPCs)	CPC-1	(35.85) ± 5.33	(40.42) ± 3.25	(48.85) ± 2.75	(71.68) ± 2.80	(79.46) ± 3.65	(67.12) ± 4.12
	CPC-2	(72.68) ± 3.38	(75.12) ± 3.55	(86.05) ± 2.90	(90.57) ± 4.09	(78.62) ± 4.22	(73.59) ± 3.10

	LSD 5%	4.81*	2.36*	2.19*	3.30*	4.32*	3.22*

*Significant at 5% level.

**Table 4:** Mean diametral tensile strength and standard deviation of hydroxyapatite cement and the two polymeric calcium phosphate cements (CPCs) [the value in parenthesis are in MPa].

Cement Types		30 min	1 hour	4 hrs	24 hrs	1 week	2 weeks
Control Hydroxyapatite cement		(1.56) ± 0.18	(1.86) ± 0.30	(1. 96) ± 0.40	(1.36) ± 0.10	(2.99) ± 0.80	(1.51) ± 0.20

Calcium phosphate Cements (CPCs)	CPC-1	(8.57) ± 2.84	(11.43) ± 2.41	(12.38) ± 2.04	(13.81) ± 2.36	(15.63) ± 2.84	(13.77) ± 2.89
	CPC-2	(12.94) ± 1.99	(15.75) ± 1.44	(16.31) ± 2.02	(21.12) ± 2.99	(17.96) ± 2.17	(17.28) ± 2.89

	LSD 5%	2.72*	2.72*	2.65*	2.63*	2.64*	2.84*

*Significant at 5% level.

**Table 5:** Scores determined for each histological feature according to the experimental and control groups and periods of evaluation.

(*n* = 6)	7 Days			30 Days			90 Days		
	CPC-1	CPC-2	HA	CPC-1	CPC-2	HA	CPC-1	CPC-2	HA
Inflammatory reaction									
None	0	0	0	0	0	0	3	5	3
Mild	0	1	2	3	4	3	2	1	2
Moderate	5	4	3	3	2	3	1	0	1
Severe	1	1	1	0	0	0	0	0	0
Fibrous capsule									
Thin	0	0	0	2	3	3	5	5	4
Thick	6	6	6	4	3	3	1	1	2
Macrophages/giant cells									
None	0	0	0	1	2	1	5	5	4
Mild	4	5	5	4	4	4	1	1	2
Moderate	2	1	1	1	0	1	0	0	0
